# TGF-*β* signaling controls FSHR signaling-reduced ovarian granulosa cell apoptosis through the SMAD4/miR-143 axis

**DOI:** 10.1038/cddis.2016.379

**Published:** 2016-11-24

**Authors:** Xing Du, Lifan Zhang, Xinyu Li, Zengxiang Pan, Honglin Liu, Qifa Li

**Affiliations:** 1 College of Animal Science and Technology, Department of Animal Genetics, Breeding and Reproduction, Nanjing Agricultural University, Nanjing, China

## Abstract

Follicle-stimulating hormone receptor (FSHR) and its intracellular signaling control mammalian follicular development and female infertility. Our previous study showed that FSHR is downregulated during follicular atresia of porcine ovaries. However, its role and regulation in follicular atresia remain unclear. Here, we showed that FSHR knockdown induced porcine granulosa cell (pGC) apoptosis and follicular atresia, and attenuated the levels of intracellular signaling molecules such as PKA, AKT and p-AKT. FSHR was identified as a target of miR-143, a microRNA that was upregulated during porcine follicular atresia. miR-143 enhanced pGC apoptosis by targeting FSHR, and reduced the levels of intracellular signaling molecules. SMAD4, the final molecule in transforming growth factor (TGF)-*β* signaling, bound to the promoter and induced significant downregulation of miR-143 *in vitro* and *in vivo*. Activated TGF-*β* signaling rescued miR-143-reduced FSHR and intracellular signaling molecules, and miR-143-induced pGC apoptosis. Overall, our findings offer evidence to explain how TGF-*β* signaling influences and FSHR signaling for regulation of pGC apoptosis and follicular atresia by a specific microRNA, miR-143.

Follicle-stimulating hormone receptor (FSHR), a member of the rhodopsin-like G protein-coupled receptor family, is specifically expressed on granulosa cells (GCs) of ovaries and Sertoli cells of testes. In ovaries of mammals, FSHR interacts with its ligand follicle-stimulating hormone (FSH) and has a crucial role in follicular development, steroidogenesis, and female infertility.^1, 2, 3, 4^ In pigs, Sato *et al.*^5, 6^ detected a quantitative trait loci (QTL) for the number of corpora lutea on SSC3 with genome-wide significance in an F2 resource population by crossing a Duroc boar with a Meishan sow, and further identified FSHR as a functional gene for this QTL by fine mapping, association, and mutational analyses. Maturation-dependent regulation of FSHR expression occurs in adult porcine ovaries.^7^ FSHR expression is downregulated during follicular atresia of pig ovaries, which is positively correlated with expression of CYP19A1, encoding a key aromatase enzyme in estradiol production, but is negatively correlated with progesterone levels in follicular fluid.^8^ However, the molecular mechanisms involved in the downregulation of FSHR during porcine follicular atresia are unclear.

Transforming growth factor (TGF)-*β* signaling has a key role in life processes by complexing with its own membrane serine/threonine kinase receptors TGFBR2 (type II receptor) and TGFBR1 (type I receptor), activating SMAD2/3 intracellular signaling, binding to SMAD4 in the nucleus, and then regulating transcription by inducing binding to target promoter regions termed SMAD-binding elements (SBEs). Activated TGF-*β* signaling decreases porcine GC (pGC) apoptosis, whereas inactivated TGF-*β* signaling enhances pGC apoptosis, indicating that TGF-*β* signaling is required for pGC function.^9, 10^ However, the mechanisms by which TGF-*β* signaling regulates pGC apoptosis remain unexplored. In ovarian GCs of other mammals, such as humans,^11^ mice,^12^ rats,^13^ and cows,^14^ TGF-β signaling is involved in FSHR expression and FSHR-induced cell function. Consistent with previous reports in mammals, TGF-*β* signaling cooperates with FOXL2 to regulate FSHR expression and GC function in pre-hierarchical follicles of hens.^15^

Small RNAs (sRNAs) are non-coding RNA molecules of 21–24 nucleotides that have a vital role in multiple cellular physiological processes by silencing target genes at the post-transcriptional level.^16, 17^ As an important type of sRNAs, microRNAs (miRNAs) are closely involved in mammalian ovarian GC functions such as GC apoptosis,^18, 19^ proliferation,^20, 21, 22^ and the cell cycle.^22^ Xu *et al.*^23^ demonstrated the regulatory effect of miRNAs on pGC function for the first time. Thereafter, a number of studies focused on the role of miRNAs in pGCs. However, only a few miRNAs have been found to be associated with pGC functions, such as miR-378,^23, 24^ miR-26b,^9, 19, 25^ Let-7g,^10, 26^ and miR-92a.^27^ Therefore, miRNA-mediated GC functions in pig ovaries need to be explored further.

In this study, we aimed to identify the miRNAs that target FSHR and to investigate the mechanism by which miRNAs regulate pGC function by targeting FSHR. Furthermore, we aimed to determine whether TGF-*β* signaling is involved in the regulation of FSHR expression in pGCs, as has been shown in other mammals,^11, 14^ and the effect of miRNAs targeting FSHR in this process.

## Results

### Involvement of FSHR in pGC apoptosis and follicular atresia

**Figure 1:**
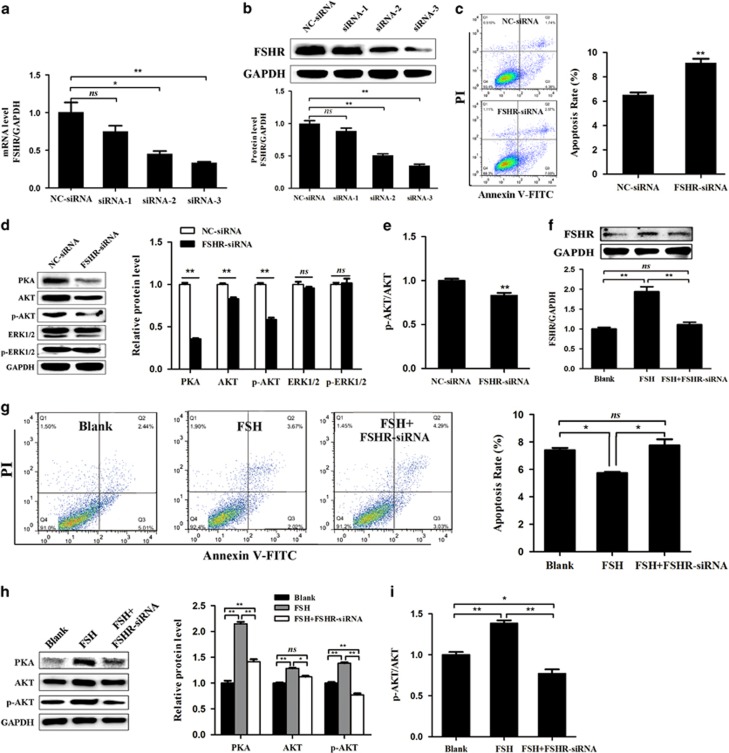
Knockdown of FSHR increases the apoptosis rate of pGCs. (**a** and **b**) Silencing of FSHR expression in pGCs using siRNAs. qRT-PCR (**a**) and western blot (**b**) analyses showed that FSHR-targeting siRNAs (siRNA-2 and siRNA-3) provided optimal depletion of FSHR in pGCs compared with negative control (NC)-siRNA. (**c**) Knockdown of FSHR increases the apoptosis rate of pGCs. pGCs transfected with NC-siRNA or FSHR-targeting siRNA were subjected to Annexin V-FITC/PI double staining and flow cytometric analysis. (**d**) The effect of FSHR knockdown on its intracellular signaling levels. (**e**) The change in the p-AKT/AKT ratio in pGCs transfected with FSHR-targeting siRNA. (**f–i**) Knockdown of FSHR inhibits FSH-mediated function. After pGCs were co-treated with FSH (6.0 IU/ml) and FSHR-targeting siRNA, western blotting was performed to measure the levels of FSHR (**f**) and intracellular signaling molecules (**h** and **i**), and flow cytometry was performed to analyze the apoptosis rate of pGCs (**g**). Average results from three independent experiments are shown. *P<0.05, **P<0.01

We previously demonstrated that FSHR is downregulated during porcine follicular atresia.^8^ To further investigate its role in follicular atresia, three FSHR-targeting small interfering RNAs (siRNAs) (siRNA-1, siRNA-2, and siRNA-3) were designed and individually transfected into pGCs. siRNA-3 had the highest knockdown efficiency at both the mRNA and protein levels in pGCs (Figures 1a and b) and was therefore used in the following experiments. Fluorescence-activated cell sorting (FACS) analysis showed that knockdown of FSHR significantly increased the apoptosis rate of pGCs (Figure 1c), implying that knockdown of FSHR can induce pGC apoptosis *in vitro*. Next, we investigated the effect of FSHR knockdown on intracellular signaling in pGCs, and showed that the protein levels of protein kinase A (PKA), AKT and p-AKT (Figure 1d) as well as the p-AKT/AKT ratio (Figure 1e) were sharply decreased, whereas expression of extracellular signal-regulated kinase 1/2 (ERK1/2) and p-ERK1/2 was not changed. In addition, silencing of FSHR attenuated FSH-induced FSHR expression (Figure 1f), FSH-reduced pGC apoptosis (Figure 1g), and FSH-induced intracellular signaling (Figures 1h and i). These results show that FSHR knockdown induces pGC apoptosis and follicular atresia, and its intracellular signaling molecules (PKA, AKT, and p-AKT) are possibly involved in this process.

### FSHR is a novel target of miR-143

**Figure 2:**
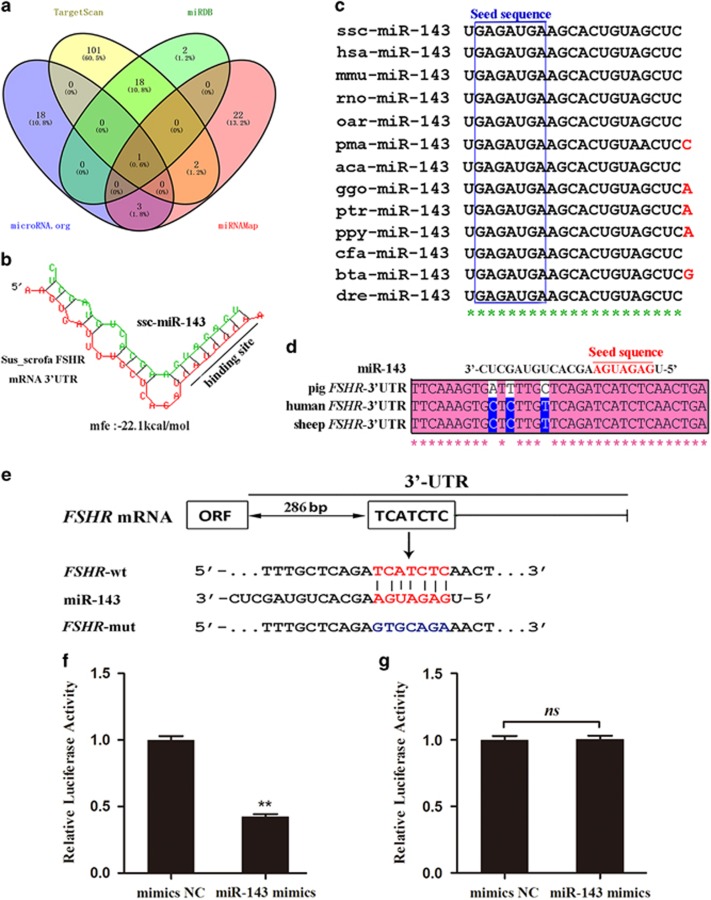
FSHR is a direct target of miR-143. (**a**) The candidate miRNAs that target FSHR were predicted by four different algorithms (TargetScan, miRDB, microRNA.org, and miRNAMap). miR-143 is the only miRNA to be predicted by all the algorithms, as shown in the Venn diagram. (**b**) The miR-143-binding site in the 3'UTR of porcine FSHR and the minimal free energy (mfe) were predicted by RNAhybrid. (**c**) miR-143 mature sequences are highly conserved among vertebrates. Nucleotides that diverge from the human sequence are highlighted in red. Asterisks indicate complementarity. ssc, *Sus scrofa*; hsa, *Homo sapiens*; mmu, *Mus musculus*; rno, *Rattus norvegicus*; oar, *Ovis aries*; pma, *Petromyzon marinus*; aca, *Anolis carolinensis*; ggo, *Gorilla gorilla*; ptr, *Pan troglodytes*; ppy, *Pongo pygmaeus*; cfa, *Canis familiaris*; bta, *Bos taurus*; dre, *Danio rerio*. (**d**) Alignments of miR-143 mature sequences and the 3'UTR of FSHR from pig, human, and sheep. Red letters indicate the seed sequences of miR-143. Asterisks indicate complementarity. (**e**) Schematic showing the interactions of miR-143 with wild-type FSHR (red) and the mutant version (blue). (**f** and **g**) Transfection of pGCs with miR-143 mimics and a dual-luciferase reporter vector containing the wild-type FSHR 3'UTR (**f**) or the mutant version (**g**). Luciferase activity was measured and normalized to that in the mimics NC group. Experiments were conducted in triplicate. **P<0.01

To explore the mechanism underlying differential expression of FSHR during porcine follicular atresia, four algorithms were used to predict the candidate miRNAs targeting FSHR. Only one miRNA (miR-143) targeting FSHR was commonly predicted by all four algorithms (Figure 2a). A putative miR-143-binding site was detected at the 286–293 nt position of the porcine FSHR 3' untranslated region (3'UTR) (Figure 2b). BLAST analysis showed that the mature sequence of pig miR-143 is highly consistent with that in other vertebrates (Figure 2c), and the seed sequence is complementary with the FSHR 3'UTR in pigs and other mammals (Figure 2d). To investigate whether miR-143 targets FSHR, we constructed a dual-luciferase reporter vector containing the wild-type miR-143-binding site located in the 3'UTR of FSHR or a mutated version (Figure 2e) and co-transfected it together with miR-143 mimics into pGCs. Luciferase activity analysis showed that miR-143 overexpression significantly decreased the luciferase activity of the wild-type reporter (Figure 2f), but had no effect on that of the mutated reporter (Figure 2g). These results suggest that FSHR is a direct target of miR-143.

### miR-143 suppresses FSHR expression in pGCs

**Figure 3:**
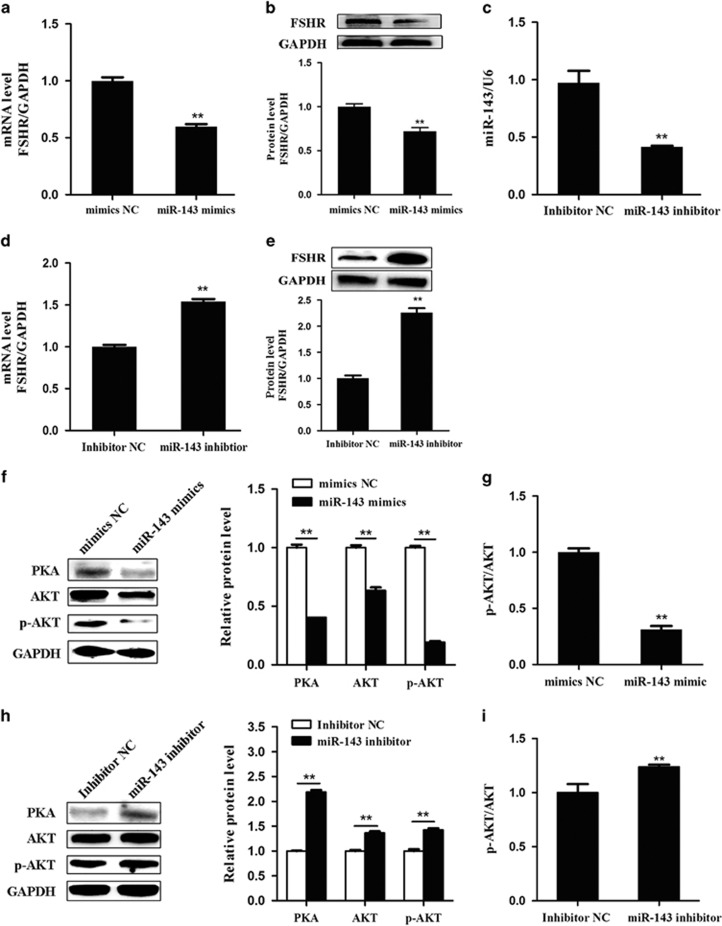
Effects of miR-143 on the expression of FSHR in pGCs. (**a** and **b**) Mimics NC or miR-143 mimics were transfected into pGCs. mRNA levels of FSHR (**a**) were detected by qRT-PCR analysis and protein levels (**b**) were detected by western blotting. (**c–e**) A specific inhibitor of miR-143 was transfected into pGCs, and miR-143 levels (**c**) were detected by miRNA qRT-PCR with normalization to that of U6. FSHR mRNA (**d**) and protein (**e**) levels were measured, and GAPDH was used as an internal control. (**f** and **g**) Overexpression of miR-143 regulates the intracellular signaling levels of FSHR (**f**) and the p-AKT/AKT ratio (**g**) in pGCs. (**h** and **i**) Silencing of miR-143 regulates the intracellular signaling levels of FSHR (**h**) and the p-AKT/AKT ratio (**i**) in pGCs. **P<0.01

We next evaluated the effect of miR-143 on FSHR expression in pGCs. Quantitative RT-PCR (qRT-PCR) and western blot analyses showed that miR-143 overexpression significantly decreased the FSHR mRNA level (Figure 3a) and protein level (Figure 3b), suggesting that miR-143 can inhibit FSHR expression in pGCs. To confirm this, a specific inhibitor of miR-143 was designed and transfected into pGCs. The miR-143 level was markedly reduced by the miR-143 inhibitor (Figure 3c). Moreover, knockdown of miR-143 significantly increased the FSHR mRNA level (Figure 3d) and protein level (Figure 3e). We also detected the protein levels of FSHR intracellular signaling molecules (PKA, AKT, and p-AKT) and showed that overexpression of miR-143 decreased their expression levels (Figure 3f) and the p-AKT/AKT ratio (Figure 3g), whereas knockdown of miR-143 significantly increased their expression levels in pGCs (Figure 3h) and the p-AKT/AKT ratio (Figure 3i). Taking these results together, we proved that miR-143 inhibits FSHR and its intracellular signaling molecules in pGCs.

### miR-143 modulates pGC apoptosis and follicular atresia by targeting FSHR

**Figure 4:**
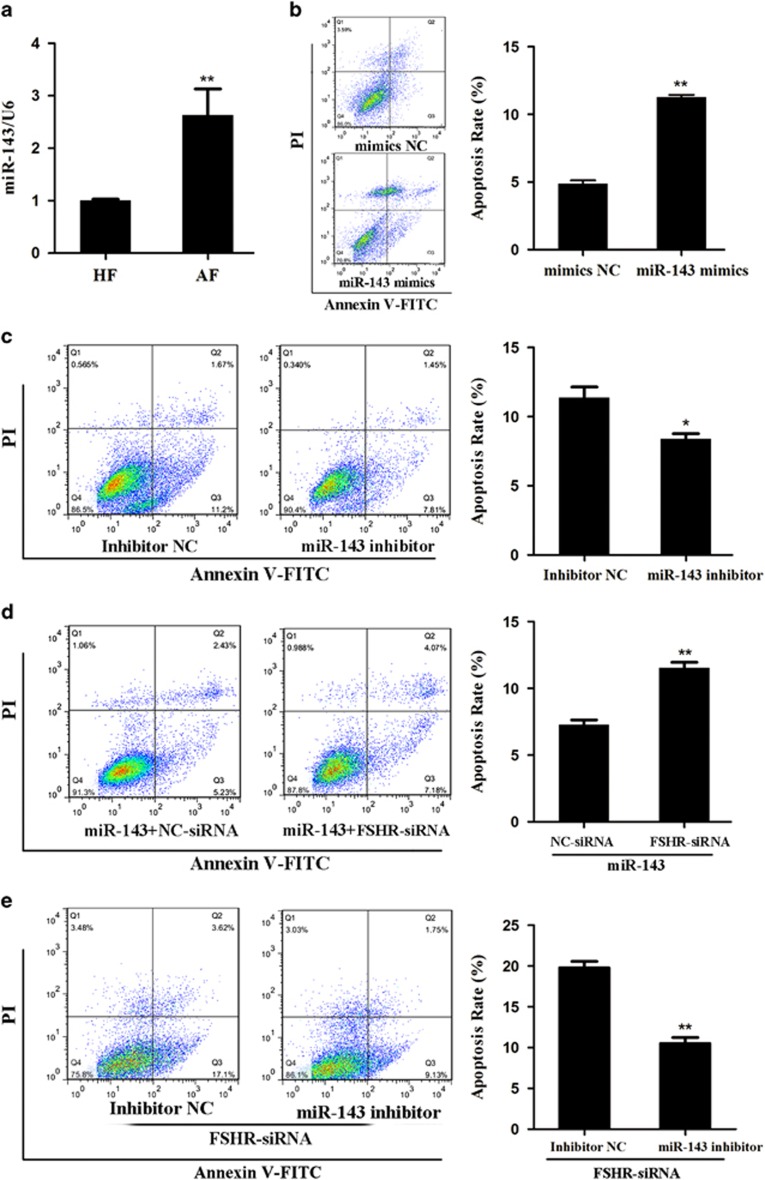
miR-143 induces pGC apoptosis by suppressing FSHR. (**a**) The expression levels of miR-143 were detected in healthy follicles (HF, *n*=6) and atretic follicles (AF, *n*=6). (**b** and **c**) The apoptosis rate was detected in pGCs transfected with miR-143 mimics (**b**) or a miR-143 inhibitor (**c**). (**d**) miR-143 mimics were co-transfected with NC-siRNA or FSHR-targeting siRNA into pGCs, and the apoptosis rate was calculated by Annexin V-FITC/PI staining and FACS. (**e**) FSHR-targeting siRNA was co-transfected with a miR-143 inhibitor or an inhibitor NC into pGCs, and the apoptosis rate was calculated. *P<0.05, **P<0.01

Our previous study showed that miR-143 is upregulated during follicular atresia using a miRNA microarray assay.^25^ qRT-PCR demonstrated that the miR-143 level was higher in atretic follicles than in healthy follicles (Figure 4a). FACS analysis revealed that miR-143 overexpression significantly increased the pGC apoptosis rate (Figure 4b), whereas knockdown of miR-143 decreased the pGC apoptosis rate (Figure 4c), suggesting that miR-143 can promote pGC apoptosis. To determine whether FSHR mediates miR-143-induced pGC apoptosis, pGCs were co-transfected with miR-143 mimics and FSHR-targeting siRNA. This showed that silencing of FSHR could increase miR-143-induced pGC apoptosis (Figure 4d). In addition, inhibition of miR-143 rescued FSHR-targeting siRNA-induced pGC apoptosis (Figure 4e). These results reveal that miR-143 regulates pGC apoptosis and follicular atresia by targeting FSHR.

### SMAD4 inhibits miR-143 expression by directly binding to its promoter

**Figure 5:**
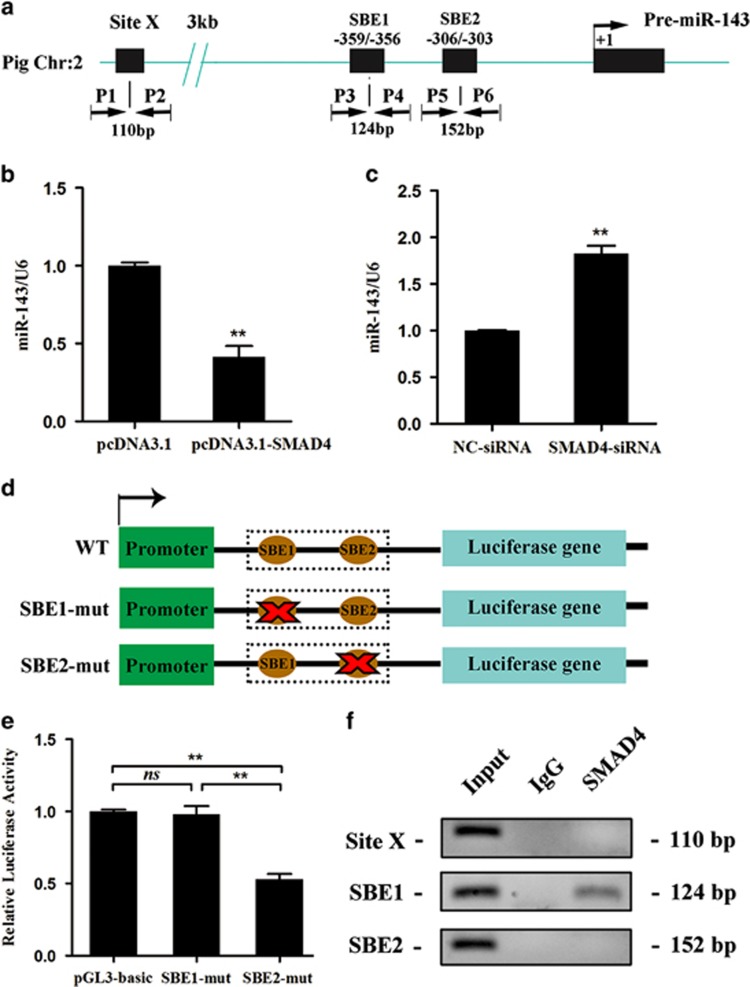
SMAD4 inhibits miR-143 expression in pGCs by binding to its promoter *in vitro* and *in vivo*. (**a**) A schematic diagram of the miR-143 promoter region. P1–P6 are the primers used for the ChIP assay. Nucleotide numbering is relative to +1 at the transcriptional start site. SBE, SMAD4-binding sites. Sites X, negative control locus for the ChIP assay. (**b** and **c**) SMAD4 regulates miR-143 expression in pGCs. SMAD4 was overexpressed (**b**) or knocked down (**c**) in pGCs and miR-143 levels were measured, with U6 as the endogenous control. (**d**) The luciferase reporter vector of the pig miR-143 promoter. The miR-143 promoter with the wild-type or mutated SBE was cloned upstream of the luciferase gene. Blue box, luciferase gene; green box, pGL-3 promoter; dotted box, miR-143 promoter; brown ovals, SBE1 and SBE2; red forks, mutation; WT, wild-type. (**e**) Luciferase activity was analyzed after pcDNA3.1-SMAD4 was co-transfected with the luciferase reporter vector. Data are normalized to the pGL-3-basic vector. (**f**) The ChIP-qPCR assay was performed to identify the interaction between SMAD4 and the miR-143 promoter. IgG was used as a negative control. Site X was used as a negative control locus. Input, total RNA was reverse-transcribed before incubation with the anti-SMAD4 antibody and amplified with primers (P1–P6). Triplicate samples were analyzed for each treatment, and the results are presented as the mean±S.E.M. *P<0.05, **P<0.01

Two SMAD4-binding sites were found at positions –359/–356 nt and –306/–303 nt in the promoter of the miR-143 gene (Figure 5a), indicating that the transcription factor SMAD4 regulates miR-143 expression. To clarify this, we investigated the effect of SMAD4 on miR-143 expression in pGCs. Overexpression of SMAD4 significantly decreased miR-143 levels (Figure 5b), whereas knockdown of SMAD4 significantly increased expression of miR-143 (Figure 5c). Furthermore, luciferase reporter vectors containing the wild-type or mutated SBE were constructed (Figure 5d) and transfected with pcDNA3.1-SMAD4 into pGCs. Luciferase activity analysis found that overexpression of SMAD4 decreased the luciferase activity of the wild-type SBE and SBE2-mut constructs, but had no effect on the SBE1-mut construct, suggesting that SMAD4 inhibits miR-143 promoter activity by binding to SBE1 in its promoter (Figure 5e). A chromatin immunoprecipitation (ChIP) assay confirmed that SMAD4 bound to –359/–356 nt in the promoter of miR-143 (Figure 5f). All these results reveal that SMAD4 regulates miR-143 expression by directly binding to its promoter *in vitro* and *in vivo*.

### SMAD4 positively regulates FSHR expression by targeting miR-143

**Figure 6:**
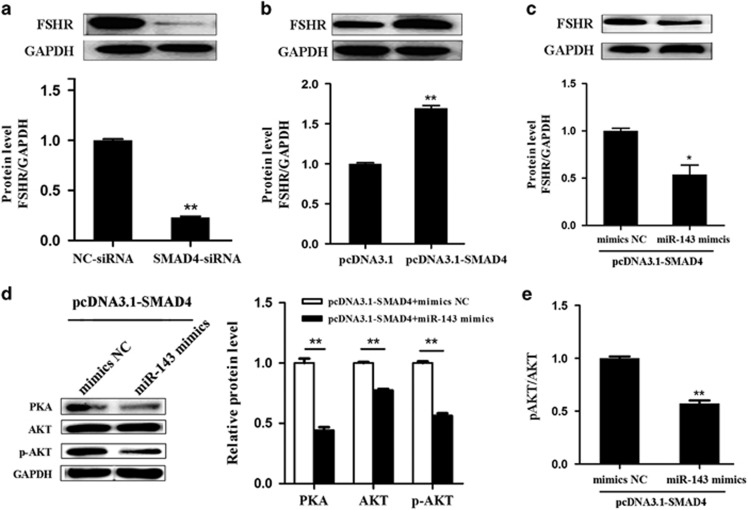
SMAD4 regulates FSHR expression via miR-143 in pGCs. (**a** and **b**) SMAD4 regulates the FSHR protein level in pGCs. Western blot analyses were conducted to detect the FSHR protein level in pGCs transfected with SMAD4-targeting siRNA (**a**) or pcDNA3.1-SMAD4 (**b**). (**c–e**) miR-143 mediates SMAD4 regulation of FSHR and intracellular signaling. miR-143 mimics were co-transfected with pcDNA3.1-SMAD4 into pGCs. The protein levels of FSHR (**c**), PKA, AKT, and p-AKT (**d**) were measured by western blotting, and the p-AKT/AKT ratio (**e**) was calculated. *P<0.05, **P<0.01

We further examined the effect of SMAD4 on the miR-143 target FSHR in pGCs. Western blotting revealed that the FSHR protein level was significantly upregulated by SMAD4 overexpression (Figure 6a) and downregulated by knockdown of SMAD4 (Figure 6b), indicating that SMAD4 positively regulates FHSR expression in pGCs. To evaluate whether miR-143 mediates this process, we co-transfected pcDNA3.1-SMAD4 and miR-143 mimics. miR-143 significantly inhibited SMAD4-induced FSHR expression (Figure 6c). In addition, miR-143 significantly decreased SMAD4-induced expression of FSHR intracellular signaling molecules (PKA, AKT, and p-AKT) (Figure 6d) and the p-AKT/AKT ratio (Figure 6e). All the results are in line with the notion that SMAD4 positively regulates FSHR expression by targeting miR-143 in pGCs.

### TGF-*β* signaling regulates the miR-143/FSHR axis in pGCs

**Figure 7:**
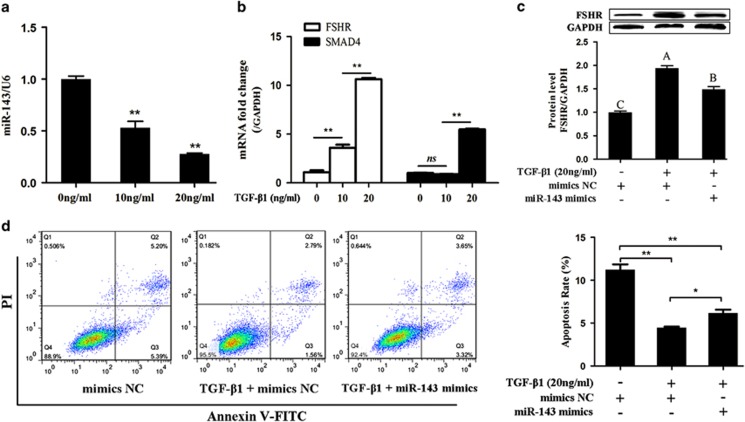
Effects of TGF-*β*1 on the expression of miR-143 and FSHR. (**a**) TGF-*β*1 suppresses the expression of miR-143. qRT-PCR analysis was performed to determine the expression of miR-143 when pGCs were treated with different concentrations of TGF-*β*1 (0, 10, and 20 ng/ml). (**b**) The SMAD4 and FSHR expression levels in pGCs are increased by TGF-*β*1 in a dose-dependent manner. (**c**) miR-143 reduces TGF-*β*1-induced expression of FSHR. pGCs were treated with 20 ng/ml TGF-*β*1 and mimics NC or miR-143 mimics, and then western blotting was performed. (**d**) miR-143 increases TGF-*β*1 reduced apoptosis of pGCs. pGCs were treated with 20 ng/ml TGF-*β*1 and mimics NC or miR-143 mimics, followed by Annexin V-FITC/PI double staining. *P<0.05; **P<0.01

SMAD4 is the final core member of the TGF-*β* signaling pathway. To determine whether SMAD4 downstream of the miR-143/FSHR axis is regulated by TGF-*β* signaling, pGCs was treated with different concentrations of TGF-*β*1 (0, 10, and 20 ng/ml) to activate TGF-*β* signaling. qRT-PCR analysis showed that the miR-143 level was suppressed sharply by TGF-*β*1 in a dose-dependent manner (Figure 7a). The mRNA levels of FSHR and SMAD4 were significantly elevated in TGF-*β*1-treated pGCs (Figure 7b). Similarly, the FSHR protein level was significantly increased in pGCs treated with TGF-*β*1 (Figure 7c). In addition, miR-143 inhibited TGF-*β*1-induced FSHR expression in pGCs (Figure 7c) and TGF-*β*1 reduced pGC apoptosis (Figure 7d). These results showed that TGF-*β* signaling controls miR-143/FSHR axis-induced apoptosis in pGCs.

## Discussion

In mammalian ovaries, the FSH-specific transmembrane receptor FSHR is specifically located on GCs, is a common marker gene of GCs, and is an important modulator of GC function. Activation of FSHR signaling strengthens GC proliferation and function,^11, 18, 28^ whereas silencing of FSHR signaling weakens GC proliferation and function.^29, 30^ Our previous study showed that FSHR is downregulated during porcine follicular atresia.^8^ In this study, we further demonstrated that FSHR can control pGC apoptosis and follicular atresia. Moreover, silencing of FSHR decreased the levels of its intracellular signaling molecules such as PKA,^11^ AKT,^30^ and p-AKT in pGCs. In GCs, binding of FSH leads to the activation of FSHR, which can activate intracellular signaling. A recent report demonstrated that enhancement of the FSHR/PKA signaling pathways should promote pGC proliferation.^28^ These results suggested that silencing of FSHR enhances apoptosis, and attenuates the levels of its intracellular signaling molecules, such as PKA, AKT, and p-AKT.

As a key gonadotropin hormone receptor and a potential target for the treatment of fertility disorders, regulation of FSHR transcription has been extensively analyzed, especially its 5' regulatory region.^31, 32, 33^ A number of putative binding sites for transcription factors were detected in the 5' regulatory region of mammalian FSHR genes. Some of these transcription factors, such as GATA-1,^34^ E2F,^34^ SF-1,^35^ and USF-1,^36^ regulate FSHR expression by binding to its 5' regulatory region. In addition, metastasis-associated protein 2, a component of histone deacetylase, can recruit histone deacetylase-1 to the FSHR promoter and participates in the downregulation of FSHR expression upon FSH treatment.^31^ In pGCs, we recently showed that histone H3K9 acetylation regulates expression of the FSHR gene.^33^ However, transcriptional regulation of its 3'UTR remains unclear. In this study, we identified miR-143 as a novel miRNA targeting FSHR and demonstrated that miR-143 inhibits FSHR expression and function in pGCs by directly binding to its 3'UTR. This result strongly suggested that the 3'UTR has an essential role in the regulation of FSHR transcription.

miR-143 is an intronic miRNA, is a member of the miR-143/145 cluster, and is co-transcribed from the same gene as miR-145.^37^ Accumulating evidence shows that miR-143 is involved in the regulation of various cellular processes, such as normal cell apoptosis, proliferation, differentiation, migration, and self-renewal,^38, 39, 40^ as well as cancer cell apoptosis, proliferation, invasion, and autophagy.^41, 42, 43, 44^ Notably, miR-143 has been extensively studied in cell apoptosis. Some apoptosis-related genes have been identified as miR-143 targets in multiple cell types, such as BCL-2 in gastric cancer cells^45^ and osteosarcoma cells,^44^ BAG3 (BCL-2-associated athanogene 3) in glioblastoma stem cells,^46^ and FSCN1 in esophageal squamous cell carcinoma.^47^ However, the role of miR-143 in GC apoptosis remains unknown. In this study, we demonstrated that miR-143 is a pro-apoptotic factor and induces pGC apoptosis and follicular atresia.

As an important signaling pathway in the body, TGF-*β* signaling may have a core role via crosstalk with other signaling pathways such as the FSHR signaling pathway.^11^ In this study, we showed that TGF-*β* signaling enhanced FSHR expression and its intracellular signaling, consistent with previous reports in other mammals.^11, 12, 48^ Addition of TGF-*β* increased FSHR mRNA expression in a time-dependent manner in rat GCs,^48^ whereas TGF-*β*1 in the presence of FSH decreased FSHR expression in bovine GCs.^14^ SMAD3, a core component of TGF-*β* signaling, is a signaling intermediate for TGF-*β*. Activated SMAD3-mediated signaling can increase expression of FSHR and its intracellular signaling,^11, 13^ and also potentiates the ovarian response to FSH stimulation in GCs.^12^ These results indicated that TGF-*β* signaling is involved in FSHR expression and FSHR-reduced pGC apoptosis.

**Figure 8:**
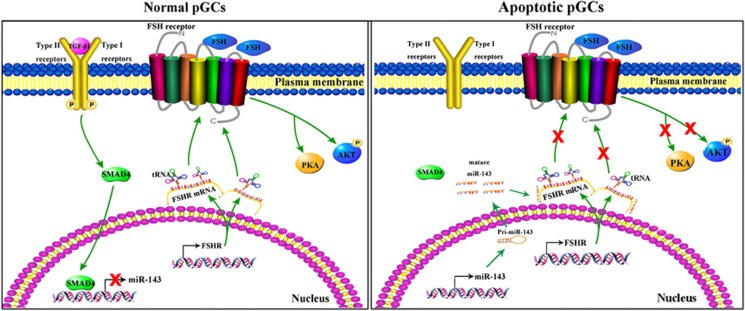
Model of crosstalk between TGF-*β* signaling and FSHR signaling for regulation of pGC apoptosis

SMAD4, as an important transcription factor as well as the final core molecule in TGF-*β* signaling, has a pivotal role in TGF-*β* signal transduction.^49, 50^ SMAD4 can complete extracellular TGF-*β* signal transmission by combining with SBEs of target promoters.^51, 52^ TGF-*β* signaling has an important role during epithelial–mesenchymal transition. Specifically, binding of SMAD4 to the SBE within the promoter region of the CAR gene in breast epithelial cells^50^ and the CDH2 gene in human pancreatic ductal epithelium^53^ and non-small cell lung cancer cells^51^ is necessary for TGF-*β*-stimulated transcription. Activation of hepatic stellate cells (HSCs) by TGF-*β*1 initiates hepatitis B virus-associated fibrogenesis. It has been recently reported that the TGF-*β*1-activated SMAD4 complex may upregulate CD147 expression by directly interacting with a specific SBE in the CD147 promoter, thereby controlling HSC migration.^52^ Here, we identified two SBEs in the miR-143 promoter and demonstrated that SMAD4 decreased miR-143 expression in pGCs by binding to SBE2 in its promoter. Interestingly, a recent study demonstrated that blockade of TGF-*β* signaling decreases the miR-143 level in smooth muscle cells.^38^ These data demonstrated that SMAD4 inactivates transcription by directly interacting with the miR-143 promoter and mediates TGF-*β* signaling-regulated FSHR expression together with miR-143. Meanwhile, our data also established the link between TGF-*β* signaling and FSHR in GCs of mammalian ovaries (Figure 8).

In conclusion, our data provide direct evidence that FSHR is an anti-apoptotic factor in pGCs and its expression and intracellular signaling are decreased by miR-143. Meanwhile, the miR-143/FSHR axis, which controls pGC function, is regulated by TGF-*β* signaling. A potential mechanism underlying regulation of the miR-143/FSHR axis by TGF-*β* signaling is that SMAD4, a central component of the TGF-*β* signaling pathway, directly binds to the SBE in the miR-143 promoter. These findings provide novel insights into the mechanism underlying GC apoptosis, follicular atresia, and follicular development in ovaries of mammals including humans.

## Materials and Methods

### Reagents

Porcine TGF-*β*1 was purchased from R&D Systems (Minneapolis, MN, USA). Antibodies against PKA, ERK1/2, and p-ERK1/2 were obtained from Sangon Biotech (Shanghai, China), those against AKT and p-AKT were from Cell Signaling Technology (Beverly, MA, USA), and those against FSHR and GAPDH as well as anti-rabbit and anti-mouse IgG were from Santa Cruz Biotechnology (Santa Cruz, CA, USA). DMEM-F-12, fetal bovine serum (FBS), and phosphate-buffered saline were obtained from Life Technologies (Carlsbad, CA, USA). Lipofectamine 2000 was purchased from Invitrogen (Carlsbad, CA, USA). Protease and phosphatase inhibitors were obtained from Roche (Basel, Switzerland). miR-143 mimics, miR-143 inhibitors, and siRNAs were all synthesized by GenePharma (Shanghai, China) (Supplementary Table S1).

### Cell culture

Fresh porcine ovaries were obtained and transported back to the laboratory within 1 h. pGC collection and pGC and 293 T cell culture were performed as previously described.^9^ All animal experiments were approved by the Animal Ethics Committee at Nanjing Agricultural University, China.

### Cell treatments and transfection

pGCs were washed with phosphate-buffered saline and cultured in serum-free DMEM/F-12 containing 6.0 IU/ml FSH (Ningbo Hormone Co., Ningbo, China) for 36 h. For transfection, cells were seeded in six-well plates. After 12 h, transfection was performed using Lipofectamine 2000 according to the manufacturer's protocol.

### RNA isolation and qRT-PCR

Total RNA was isolated from pGCs using a High Purity Total RNA Extraction Kit (Bioteke, Beijing, China) and was then reverse-transcribed into cDNA using PrimeScript™ RT Master Mix (TaKaRa, Dalian, China). qRT-PCR was performed using SYBR Green Master Mix (Vazyme Biotech, Nanjing, China) and relative expression levels were calculated using the 2^-ΔΔCt^ method. The primers used are listed in Supplementary Table S2. The expression level of mature miR-143 was measured using a PrimeScript^®^ miRNA qPCR Starter Kit (TaKaRa). GAPDH and U6 small nuclear RNA were used as endogenous controls for genes and miRNAs, respectively.

### Western blotting

Total protein was extracted from pGCs using radioimmunoprecipitation assay buffer containing 1% phosphatase inhibitor (v/v). The protein concentration was determined by the BCA method (Pierce, Shanghai, China). A 12% SDS-PAGE gel was prepared and 25 *μ*g of protein was loaded. After electrophoresis for 1 h, the total protein was transferred to a PVDF membrane (Millipore, Billerica, MA, USA). The membrane was blocked in 5% non-fat milk and then incubated with primary antibodies overnight at 4 °C. After washing, the appropriate secondary antibodies were used and chemiluminescence was detected.

### Apoptosis analysis

The apoptosis rate of pGCs was measured using an Apoptosis Detection Kit (Vazyme Biotech) according to the manufacturer's protocol. In total, 2 × 10^5^ cells were sorted by FACS using a cell counting machine (Becton Dickinson, Franklin, NJ, USA). The apoptosis rate was calculated using the following equation: (number of cells in the right upper quadrant+number of cells in the right lower quadrant)/(total number of cells).

### Bioinformatics analysis

miRNAs that target FSHR were predicted by four online software programs, TargetScan (http://www.mirbase.org/), miRDB (http://www. mirdb.org/miRDB/), microRNA.org (http://www.microrna.org/), and miRNAMap (http://mirnamap.mbc.nctu.edu.tw/). RNAhybrid (http://bibiserv.techfak.uni–bielef eld.de/rnahybrid/) was used to predict the miR-143-binding sites in the 3'UTR of FSHR. miR-143 mature sequences of different species were obtained from miRBase (http://www.mirbase.org/).

### Plasmid construction

For miR-143-binding site detection, the pmirGLO Dual-luciferase miRNA Target Expression Vector containing the wild-type FSHR 3'UTR was constructed. For SMAD4-binding site detection, the pGL-3 reporter vector containing the wild-type miR-143 promoter was constructed. Mutant plasmids were constructed using a TaKaRa MutanBEST Kit (TaKaRa) according to the manufacturer's instructions. The pcDNA3.1-SMAD4 plasmid was prepared previously by our group.^9^

### Luciferase reporter assay

After transfection for 24 h, pGCs were collected and luciferase activities were measured using the Dual-Luciferase Reporter Assay System (Promega, Madison, WI, USA) according to the manufacturer's instructions.

### Chromatin immunoprecipitation

In total, 1 × 10^7^ pGCs were fixed in 1% formaldehyde and sonicated for 5 min (10-s on and 10-s off) on ice with a 3 mm microtip and the output control set to 30%. The sonicated chromatin fluid (800 *μ*l) was collected by centrifugation. Then, sonicated chromatin (400 *μ*l) was diluted 2.5-fold and mixed with protein A/G-agarose (40 *μ*l), followed by shaking at 4 °C for 1 h. This mixture was then centrifuged at 5000 r.p.m. for 1 min, and the supernatant was collected into a new dolphin tube. An anti-SMAD4 antibody and rabbit IgG (2 *μ*g) were added and the sample was incubated overnight at 4 °C with shaking. After crosslinking reversal and proteinase K treatment, DNA was released, precipitated, and diluted in 50–100 μl of 10 mM Tris-HCL (pH 8.0). PCR-amplified products were analyzed on a 3% agarose gel.

### TGF-*β*1 treatment

pGCs were seeded in six-well plates at a density of 1 × 10^6^ cells per well and cultured in medium containing 10% FBS for 24 h. Then, the medium was replaced with serum-free medium and TGF-*β*1 was added to a final concentration of 10 and 20 ng/ml. After 24 h, cells were collected and the expression levels of miR-143, SMAD4, and FSHR were determined.

### Statistical analysis

Statistical analysis was performed using IBM SPSS Statistics v20.0 (SPSS Inc., Chicago, IL, USA). An unpaired two-sided Student's *t*-test and a one-way analysis of variance were used to evaluate the significance of statistics. *P*<0.05 was considered statistically significant. All data are presented as means±S.E.M.
