# Supplementary material for: TGF-β signaling controls FSHR signaling-reduced ovarian granulosa cell apoptosis through the SMAD4/miR-143 axis
Source: Cell Death Dis. 2016 Nov 24;7(11):e2476–. doi: 10.1038/cddis.2016.379 (PMC5260897; doi:10.1038/cddis.2016.379)
Supplement: Supplementary Tables [file cddis2016379x1.doc]

**SUPPLEMENTAL DATA LEGENDS**

**Table S1 Small fragments of RNA synthesized in the present study**

**Table S2 Primers used in this study.**

**Table S1**

| **Name** | **Sequences** |
| --- | --- |
| MiR-143 mimics | 5’-UGAGAUGAAGCACUGUAGCUC-3’ |
| Mimics NC | 5’-UUGUACUACACAAAAGUACUG-3’ |
| MiR-143 inhibitor | 5’-GAGCUACAGUGCUUCAUCUCA-3’ |
| Inhibitor NC | 5’-CAGUACUUUUGUGUAGUACAA-3’ |
| FSHR-siRNA-1 | 5’-CCAACAACCUGCUUUACAUUU-3’ |
|  | 5’-AUGUAAAGCAGGUUGUUGGTT-3’ |
| FSHR-siRNA-2 | 5’-GCAAAUCUCUGACCUUCAUUU-3’ |
|  | 5’-AUGAAGGUCAGAGAUUUGCTT-3’ |
| FSHR-siRNA-3 | 5’-GCCGAUCUCUGCAUUGGAAUU-3’ |
|  | 5’-UUCCAAUGCAGAGAUCGGCTT-3’ |
| SMAD4-siRNA | 5’-CACCAGGAAUUGAUCUCUCAGGAUU-3’ |
|  | 5’-AAUCCUGAGAGAUCAAUUCCUGGUG-3’ |
| NC siRNA | 5’-GCGACGAUCUGCCUAAGAUdTdT-3’ |
|  | 5’-AUCUUAGGCAGAUCGUCGCdTdT-3’ |

**Table S2**

| **Gene** | **Primer sequences** | **Product size (bp)** | **Tm (°C)** | **Usage** |
| --- | --- | --- | --- | --- |
| FSHR | F: 5’-CTTGCCTTTGAAGACTATGCC-3’  R: 5’-AGAACCCCAAAAGGTCAAACT-3’ | 803 | 57.3 | 3’ UTR vector construction |
| MiR-143 | F: 5’-TGTGCTGTTTGACTGGGATG-3’  R: 5’-GCCGTGGTCACAGTCAGTTT-3’ | 207 | 57.9 | Promoter vector  construction |
| MiR-143 | F: 5’-CTCCGTCCCCTGAAAT*TCTT*CCAGGCAGCTCTAG-3’  R: 5’-CCACCCCACAGCATGTTCGTGCTGCTTCTATT-3’ | 5025 | 59.0 | SBE1 mutant  vector construction |
| MiR-143 | F: 5’-GGGTTCTTTTGGACCCTGAAATGTGCTAAAAC-3’  R: 5’-TCTTACCGTGT*ATC*CCCCCTTCCCTCCCC-3’ | 5025 | 59.5 | SBE2 mutant  vector construction |
| MiR-143 | F: 5’-TGCTGTTTGACTGGGATGTG-3’  R: 5’-TGGGCAGAGCGGTCTCTCTA-3’ | 124 | 60.0 | SBE1  ChIP-qPCR |
| MiR-143 | F: 5’-GGTCTACACGGTAAGAGGGTTCT-3’  R: 5’-TTCCCTTCCGAGCAGTGTTAT-3’ | 152 | 60.0 | SBE2  ChIP-qPCR |
| MiR-143 | F: 5’-CCCCTATTCGCCTTGCCAAG-3  R: 5’-CAAACCGAACGCATCGCCTG-3 | 110 | 55.2 | X  ChIP-qPCR |
| FSHR | F: 5’-AGTCCCTCGGTTCCTTAT-3’  R: 5’-CATCTTTCCAGGGTGAT-3’ | 219 | 57.0 | qRT-PCR |
| SMAD4 | F: 5’-ATTGGTGTTCCATTGCCTAC-3’  R: 5’- TGGTCACTAAGGCACCTGAC-3’ | 250 | 58.0 | qRT-PCR |
| GAPDH | F: 5’-GATGGTGAAGGTCGGAGTG-3’  R: 5’-CGAAGTTGTCATGGATGACC-3’ | 226 | 58.0 | qRT-PCR |
| MiR-143 | 5’-GCGTGAGATGAAGCACTGTAGCTC-3’ |  |  | miRNA qRT-PCR |
